# Evaluation of the Safety and Efficacy of Ganga Water-Phage Therapy Plus Standard Medical Therapy Compared to Standard Medical Therapy Alone for Helicobacter pylori-Related Dyspepsia: A Randomized Controlled Trial

**DOI:** 10.7759/cureus.97383

**Published:** 2025-11-20

**Authors:** Virendra Verma, Archana Devi, Bharat Jhunjhunwala, Ravi Kumar, Jitendra Singh, Mahesh Chandra Pandey, Ajay Patwa

**Affiliations:** 1 General Medicine, Rajarshi Dashrath Autonomous State Medical College, Ayodhya, IND; 2 Gastroenterology and Hepatology, King George's Medical University, Lucknow, IND; 3 Medicine, Motilal Bhimraj Charity Trust, Kanpur, IND; 4 General Medicine, King George's Medical University, Lucknow, IND; 5 General Surgery, Rajarshi Dashrath Autonomous State Medical College, Ayodhya, IND; 6 Medicine, Gastroenterology and Hepatology, King George’s Medical University, Lucknow, IND

**Keywords:** adjunct therapy, dyspepsia, ganga water-phage therapy, helicobacter pylori, quality of life, randomized controlled trial, symptom relief

## Abstract

Background: Dyspepsia is a prevalent gastrointestinal condition in India, commonly linked to *Helicobacter pylori* infection. Standard medical therapy (SMT) with proton-pump inhibitors and antibiotics shows declining success due to rising antimicrobial resistance. Ganga water-phage therapy (GWPT), rich in natural bacteriophages with reported antibacterial effects, may offer a culturally relevant adjuvant strategy.

Objective: This study aimed to evaluate the safety and efficacy of GWPT combined with SMT versus SMT alone in *H. pylori*-related dyspepsia.

Methods: This prospective, randomized, controlled, open-label clinical trial was conducted between March 2025 and July 2025 at two tertiary-care centers in northern India: Rajarshi Dashrath Autonomous State Medical College, Ayodhya, and King George’s Medical University (KGMU), Lucknow. In this randomized controlled trial (CTRI/2024/07/070007), 50 adults aged 18-65 years with confirmed *H. pylori* infection (rapid urease test positive) were randomized to receive either SMT alone (n = 24) or SMT plus GWPT (n = 26) for 14 days. Outcomes included *H. pylori* eradication at two weeks, symptom improvement using the Gastrointestinal Distress Scale (GOS), quality of life via SF-36, and safety assessment. Statistical analyses used t-tests, Mann-Whitney U, chi-square, and Wilcoxon signed-rank tests with significance set at p < 0.05.

Results: Eradication rates were 66.7 % for SMT and 69.2 % for SMT + GWPT (p = 1.000). Both groups improved significantly in symptom and quality-of-life measures, with numerically greater gains in the GWPT arm. Adverse events were mild (abdominal discomfort, nausea, gastritis, and belching); no serious toxicity occurred.

Conclusion: GWPT, when added to SMT, marginally enhances *H. pylori* eradication. It confers greater improvements in symptoms and quality of life, with good safety and tolerability. GWPT may serve as a culturally acceptable adjuvant therapy in dyspepsia management. Larger multicenter studies with longer follow-up and antimicrobial resistance profiling are warranted.

## Introduction

*Helicobacter pylori *(*H. pylori*) infection continues to be a major global cause of gastritis, peptic ulcer disease, and gastric malignancy, affecting over half of the world’s population [1]. In India, its prevalence among dyspeptic patients ranges between 35% and 85% depending on regional and socioeconomic factors [2,3]. Conventional first-line triple therapy, consisting of a proton-pump inhibitor (PPI), clarithromycin, and amoxicillin or metronidazole, has shown declining eradication rates globally due to emerging antimicrobial resistance [4,5]. The growing need for novel, safe, and culturally acceptable adjuncts has generated interest in exploring natural sources with antibacterial or immunomodulatory potential.

Ganga River water has been historically regarded for its self-purifying properties. Modern microbiological investigations have attributed this phenomenon to the presence of naturally occurring bacteriophages, viruses that selectively infect and lyse bacteria [6,7]. Studies from the Himalayan and upper-Ganga regions have demonstrated that such phages exhibit broad-spectrum lytic activity against *Escherichia coli*, *Staphylococcus aureus*, *Pseudomonas aeruginosa*, and even *Vibrio cholerae* [6-8]. This scientific rationale provides a biologically plausible basis for exploring whether Ganga water-phage therapy (GWPT) could assist in bacterial modulation within the gastrointestinal tract, including *H. pylori* infection.

Functional dyspepsia and *H. pylori*-associated dyspepsia are defined under the Rome IV criteria, characterized by postprandial fullness, early satiation, epigastric pain, or burning without any structural lesion [9]. Persistent dyspeptic symptoms after standard treatment often lead to reduced quality of life and psychological distress [10-14]. Considering the emerging concept of microbiome balance and bacteriophage-mediated modulation of microbial ecology, the potential role of phage-based natural therapies warrants systematic investigation.

This randomized pilot study was designed to evaluate the safety, tolerability, and preliminary efficacy of GWPT as an adjunct to standard medical therapy in *H. pylori*-associated dyspepsia. The study aimed to assess its effect on *H. pylori* eradication rates, symptom severity, and quality-of-life improvement, while ensuring biosafety and adherence to ethical environmental use standards.

## Materials and methods

Study design and oversight

This prospective, randomized, controlled, open-label clinical trial was conducted between March 2025 and July 2025 at two tertiary-care centers in northern India: Rajarshi Dashrath Autonomous State Medical College, Ayodhya, and King George’s Medical University (KGMU), Lucknow. The objective was to evaluate the efficacy and safety of GWPT as an adjunct to standard medical therapy (SMT) in adults with *H. pylori*-associated dyspepsia. The study protocol was reviewed and approved by the Institutional Ethics Committee (Approval No. RDASMC/IEC/2023/15) and registered prospectively with the Clinical Trials Registry of India (CTRI/2024/07/070007). Written informed consent was obtained from all participants prior to enrolment. The study was conducted in full accordance with the Declaration of Helsinki (2013 revision) and the International Conference on Harmonisation-Good Clinical Practice (ICH-GCP) guidelines.

Participants

Fifty adult patients aged 18-60 years, presenting with symptoms of dyspepsia as per the Rome IV criteria and confirmed *H. pylori*-positive by rapid urease test (RUT), were enrolled after obtaining written informed consent. Patients with a history of recent antibiotic or proton-pump inhibitor use (within four weeks), gastrointestinal malignancy, chronic liver or renal disease, pregnancy, or known hypersensitivity to study drugs were excluded.

RUT was used as an initial screening tool for *H. pylori* infection. Confirmatory diagnostic tests, such as ^13^C-urea breath test or stool antigen assay, were not performed owing to the exploratory pilot design.

Randomization and intervention

Participants were randomized into two groups using a computer-generated random allocation sequence:

Group I (SMT): standard medical therapy (PPI + clarithromycin + amoxicillin) for 14 days

Group II (SMT + GWPT): standard medical therapy plus oral administration of 100 mL of GWPT twice daily for 14 days

Compliance was assessed by medication count and participant self-reporting. All participants received dietary and hygiene counseling to avoid confounding.

Preparation and quality control of GWPT

The Ganga water used in this study was collected aseptically from the upper Himalayan region (Rishikesh, Uttarakhand) in sterile glass containers and filtered through a 0.22 µm membrane. The preparation was screened microbiologically to ensure the absence of coliforms, *E. coli*, *Salmonella*, and *Pseudomonas*, and tested for total viable counts, pH, turbidity, and heavy metals according to the WHO and Central Pollution Control Board (CPCB) guidelines.

The presence of naturally occurring bacteriophages was confirmed indirectly by double-layer plaque assay using standard bacterial lawns, following previously published protocols [11,12]. The preparation was stored at 4°C and freshly dispensed every seven days.

Safety evaluation

Safety monitoring was conducted throughout the study using structured questionnaires and laboratory assessments. All adverse events (AEs) were recorded and graded according to the Common Terminology Criteria for Adverse Events (CTCAE v5.0). Severity, causality, and outcome were documented. Serious adverse events were reported to the Institutional Ethics Committee within 24 hours. In addition, an independent safety reviewer verified that the water preparation met biosafety standards prior to use.

Outcome measures

Primary Outcome

The primary endpoint was *H. pylori* eradication, assessed two weeks after completion of therapy using a repeat RUT performed during follow-up endoscopy. Eradication was defined as a negative RUT at two weeks post-treatment.

Secondary Outcomes

The secondary outcomes were changes in symptom severity using the Glasgow Dyspepsia Severity Score (GOSS) [13], changes in the quality of life assessed by the 36-Item Short Form Health Survey (SF-36) questionnaire, and incidence and severity of adverse events.

Quality of life was assessed using the SF-36, a validated tool that measures eight domains: physical functioning, role limitations due to physical health, bodily pain, general health perception, vitality, social functioning, role limitations due to emotional problems, and mental health. The SF-36 instrument has been widely used as a comprehensive measure of overall health-related quality of life [14].

Sample size calculation

The required sample size was estimated using the Z-p-d formula for two proportions. Assuming an expected difference of 25% between groups (80% vs. 55% eradication), α = 0.05, and 80% power, the calculated minimum sample size was 23 per group. To account for attrition, 25 participants were recruited per arm, yielding a total sample of 50.

Laboratory and safety assessments

Baseline and post-treatment evaluations included hematological and biochemical tests - hemoglobin, total and differential leukocyte counts, platelet indices, renal and liver function tests, serum electrolytes, and glycosylated hemoglobin (HbA1c). Adverse events were recorded through structured patient interviews and physical examinations during and after treatment. All adverse effects were graded according to their intensity and relation to the intervention.

Statistical analysis

All analyses were conducted using IBM SPSS Statistics for Windows, version 26.0 (released 2018, IBM Corp., Armonk, NY). Continuous variables were expressed as mean ± SD or median (IQR), depending on data distribution. Categorical variables were presented as frequencies and percentages. Between-group comparisons were performed using the Chi-square or Fisher’s exact test for categorical data and the Mann-Whitney U test for continuous variables. Within-group changes were analyzed using the Wilcoxon signed-rank test. All tests were two-tailed, and p < 0.05 was considered statistically significant. For variables showing non-normal distribution or wide standard deviations, data were also summarized as median with IQR. For skewed variables such as platelet counts and hematocrit, data are additionally presented as median (IQR) to account for distributional skew.

To control for multiple testing, the Benjamini-Hochberg false discovery rate (FDR) correction was applied. All analyses were performed on an intention-to-treat basis; a per-protocol sensitivity analysis yielded similar trends. Missing data were handled using the last-observation-carried-forward (LOCF) method.

Ethical considerations

The study adhered to all ethical and regulatory requirements, maintaining strict confidentiality of participant data and ensuring that all analyses were conducted on anonymized datasets. Ethical and safety compliance was upheld in accordance with the principles of the Declaration of Helsinki, the CPCB biosafety norms, and the Indian Council of Medical Research (ICMR) guidelines for human research and environmental responsibility. The study followed all ethical standards for human participation and safe handling of natural water sources, ensuring transparency, accountability, and environmental integrity throughout the research process. No external funding or commercial sponsorship was received, no financial incentives were provided to participants, and none of the investigators reported any conflict of interest.

## Results

Baseline characteristics

Values are mean ± SD, unless otherwise stated. p < 0.05 is considered significant.

**Figure 1:**
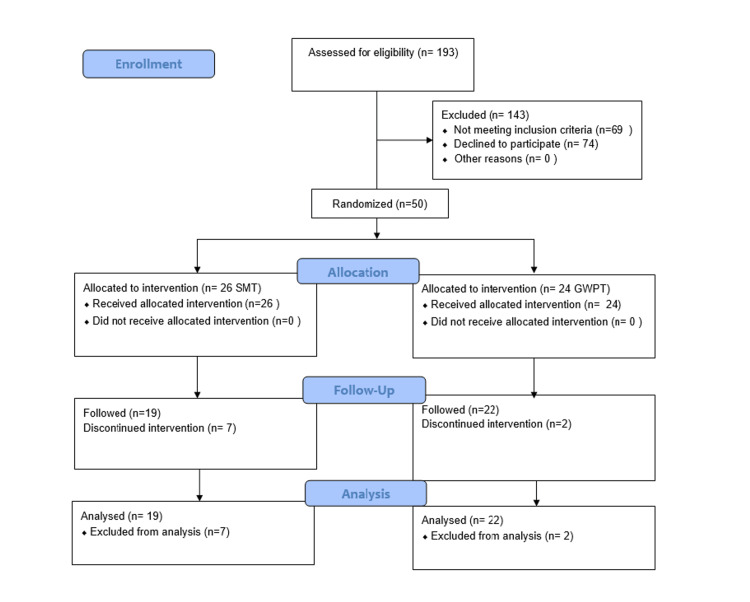
CONSORT flow diagram showing enrollment (n = 50), allocation, follow-up, and analysis.

The study flow chart is given in Figure 1. Table 1 summarizes the baseline demographic and lifestyle characteristics of the 50 enrolled participants, evenly distributed between the SMT group (n = 24) and the SMT plus GWPT group (n = 26). The mean age of the total cohort was 37.98 ± 14.11 years, with no significant difference between the SMT (37.38 ± 13.19 years) and SMT plus GWPT groups (38.54 ± 15.15 years, p = 0.774). Similarly, anthropometric measures, including the mean height (158.94 ± 7.07 cm), weight (67.03 ± 12.00 kg), and BMI (26.55 ± 4.59 kg/m²), were comparable between the two groups (p > 0.05 for all). Gender distribution was balanced, with 40% females overall (41.7% in SMT vs. 38.5% in SMT plus GWPT, p = 0.817). All participants were normotensive. Lifestyle factors also did not differ significantly: smoking was rare (2.0% overall, with only one smoker in the SMT group), tobacco chewing was reported by 2.0% overall (one case in each group), and alcohol consumption was similarly uncommon (2-4% across both groups, p > 0.05). Thus, both study arms were well matched for demographic variables and lifestyle factors at baseline, minimizing potential confounding in subsequent outcome assessments.

**Table 1:** Comparison between baseline demographic and lifestyle characteristics of participants in the SMT and SMT plus GWPT groups. *a = applied unpaired t-test. *b = c2 test/ Fisher's exact test as appropriate. Two-sample t-test, chi-square test, and Fisher’s exact tests are used to test the independence of attributes. All comparisons yielded p > 0.05, confirming that baseline demographic variables were statistically comparable between groups. SMT: standard medical therapy, GWPT: Ganga water-phage therapy

	Group						p-value
	Total (n = 50)		SMT group (n = 24)		SMT plus GWPT group (n = 26)		
	N	%	N	%	N	%	
Age (in years)	37.98 ± 14.11		37.38 ± 13.19		38.54 ± 15.15		0.774a
Height (cm)	158.94 ± 7.07		158.79 ± 6.05		159.08 ± 8.01		0.888a
Weight (kg)	67.03 ± 12.00		68.80 ± 11.60		65.38 ± 12.35		0.319a
BMI	26.55 ± 4.59		26.30 ± 3.96		26.77 ± 5.17		0.720a
Gender	20	40.0%	10	41.7%	10	38.5%	0.817b
	30	60.0%	14	58.3%	16	61.5%	
No hypertension	50	100.0%	24	100.0%	26	100.0%	NA
Smoking status	1	2.0%	1	4.2%	0	.0%	0.470b
	49	98.0%	23	95.8%	26	100%	
Tobacco chewing status	1	2.0%	1	4.2%	1	3.8%	0.515b
	49	98.0%	23	95.8%	25	96.2%	
Alcohol consumption status	1	2.0%	0	.0%	1	3.8%	0.570b
	1	2.0%	1	4.2%	0	.0%	
	2	4.0%	1	4.2%	1	3.8%	
	46	92.0%	22	91.7%	24	92.3%	

Baseline demographic and lifestyle profiles were comparable between groups, confirming successful randomization (Table 1). The mean age was 37.9 ± 14.1 years with a balanced gender distribution (40% females overall). No significant differences were observed in BMI, lifestyle factors, or comorbidities (all p > 0.05).

Table 2 presents the baseline hematological and biochemical parameters of the study participants, comparing the SMT (n = 24) and SMT plus GWPT (n = 26) groups. Hemoglobin levels were similar (12.94 ± 1.76 g/dl vs. 12.04 ± 2.08 g/dl, p = 0.257), as were the total leukocyte counts (2.05 ± 3.10 vs. 1.57 ± 3.03 ×10³/µL, p = 0.133). Platelet counts showed wide variability but no group difference (p = 0.910), and the mean platelet volume was also comparable (11.63 ± 3.78 vs. 11.51 ± 1.32, p = 0.987). Red blood cell (RBC) indices, including total RBCs, mean corpuscular volume (MCV), mean corpuscular hemoglobin (MCH), and mean corpuscular hemoglobin concentration (MCHC), were not significantly different between groups, although hematocrit values approached significance with slightly lower levels in the SMT plus GWPT group (35.77 ± 5.78 vs. 38.58 ± 7.70, p = 0.050). Platelet indices such as plateletcrit (PCT) and platelet distribution width (PDW) did not differ significantly. Differential counts for absolute neutrophils, lymphocytes, eosinophils, monocytes, and basophils were comparable, with eosinophils showing a non-significant trend toward higher values in the SMT group (p = 0.079). Renal parameters, including serum urea (20.23 ± 6.53 vs. 24.24 ± 6.32 mg/dl, p = 0.085) and creatinine (0.85 ± 0.19 mg/dl in both groups, p = 0.865), were within normal ranges. Liver function markers, such as total and direct bilirubin, serum glutamic-oxaloacetic transaminase (SGOT), and serum glutamic pyruvic transaminase (SGPT), did not differ significantly. Electrolytes, including serum sodium (139.42 ± 1.75 vs. 138.00 ± 1.22 mmol/L, p = 0.198) and potassium (4.08 ± 0.87 vs. 3.71 ± 0.47 mmol/L, p = 0.317), were stable across groups, as were serum proteins, albumin, and ionic calcium. Glycemic status assessed by HbA1c was also comparable (5.43 ± 0.78 vs. 4.76 ± 0.83, p = 0.215). Overall, baseline hematological and biochemical parameters were balanced between groups, indicating no pre-treatment disparities that could bias outcome assessment (Table 2).

**Table 2:** Distribution of patients by clinical findings Baseline haematological and biochemical parameters in the SMT and SMT plus GPT groups were based on data from 50 patients. SMT: standard medical therapy, GWPT: Ganga water-phage therapy, HB: hemoglobin, TLC: total leukocyte count, MPV: mean platelet volume, RBC: red blood cell, MCV: mean corpuscular volume, MCH: mean corpuscular hemoglobin, MCHC: mean corpuscular hemoglobin concentration, HCT: hematocrit, PCT: plateletcrit, PDW: platelet distribution width, Abs. neutrophils: absolute neutrophil count, Abs. lymphocytes: absolute lymphocyte count, Abs. eosinophils: absolute eosinophil count, Abs. monocyte: absolute monocyte count, Abs. basophil: absolute basophil count, SGOT: serum glutamic-oxaloacetic transaminase, SGPT: serum glutamic pyruvic transaminase, HbA1c: glycated hemoglobin

	Group						Z-value	p-value
	Total (n = 50)		SMT group (n = 24)		SMT plus GWPT group (n = 26)			
	Mean	SD	Mean	SD	Mean	SD		
HB (gm/dl)	12.55	1.93	12.94	1.76	12.04	2.08	-1.134	0.257
TLC (×10³/µL)	1.84	3.04	2.05	3.10	1.57	3.03	-1.501	0.133
Platelet count (×10³/µL)	6.70	40.97	11.80	54.55	0.14	0.10	-0.113	0.910
MPV	11.58	2.85	11.63	3.78	11.51	1.32	-0.016	0.987
Total RBCs	5.36	6.74	6.36	8.91	4.08	.70	-1.699	0.089
MCV (mean cell volume)	86.31	15.95	84.59	19.62	88.54	9.47	-0.567	0.571
MCH (mean corpus haemoglobin)	28.54	5.71	27.50	6.76	29.88	3.76	-1.601	0.109
MCHC (mean corpus Hb conc.)	40.59	47.79	32.48	2.29	51.10	72.17	-1.884	0.060
HCT (hematocrit)	37.21	6.88	38.58	7.70	35.77	5.78	-1.964	0.050
PCT	0.21	0.07	0.22	0.07	0.21	0.08	-1.227	0.220
PDW	15.75	4.58	15.04	5.19	16.51	3.86	-1.154	0.249
Abs. neutrophils	4.12	2.55	3.88	1.52	4.36	3.36	-0.749	0.454
Abs. lymphocytes	1.86	.58	1.97	0.54	1.74	0.61	-1.535	0.125
Abs. eosinophils	0.19	0.22	0.25	0.28	0.11	0.07	-1.754	0.079
Abs. monocyte	0.61	0.87	0.53	0.50	0.69	1.16	-0.054	0.957
Abs. basophil	0.01	0.02	0.01	0.02	0.01	0.02	-0.816	0.415
Serum urea	22.11	6.64	20.23	6.53	24.24	6.32	-1.72	0.085
Serum creatinine	0.85	0.18	0.85	0.19	0.85	0.19	-0.17	0.865
Serum bilirubin, total	0.56	0.26	0.58	0.26	0.53	0.25	-0.734	0.463
Serum bilirubin, direct	0.25	0.11	0.27	0.11	0.23	0.10	-1.146	0.252
SGOT	44.59	60.32	44.10	65.65	45.20	55.01	-0.646	0.518
SGPT	43.92	59.12	46.54	72.98	40.69	37.48	-0.073	0.941
Serum sodium	138.92	1.69	139.42	1.75	138.00	1.22	-1.287	0.198
Serum potassium	3.95	0.76	4.08	0.87	3.71	0.47	-1.001	0.317
Serum ionic calcium	1.63	1.13	1.92	1.37	1.13	0.11	-0.475	0.635
Serum proteins	7.28	0.69	7.31	0.65	7.19	0.86	-0.036	0.972
Serum albumin	4.04	0.46	4.05	0.52	4.04	0.36	-0.071	0.944
HbA1c	5.15	0.83	5.43	0.78	4.76	0.83	-1.24	0.215

All biochemical parameters were within normal physiological ranges. Minor variations, such as slightly lower hematocrit in the SMT + GWPT arm, were clinically insignificant.

*H. pylori *eradication

Table 3 shows the comparative results of the RUT at baseline and two weeks post-treatment. At enrollment, all 50 participants (100%) in both the SMT group (n = 24) and the SMT plus GWPT group (n = 26) tested positive for* H. pylori i*nfection, confirming eligibility. After two weeks of therapy, follow-up RUT was available for 41 participants, with overall eradication achieved in 34 cases (68.0%). The eradication rates were nearly identical between the groups: 66.7% in the SMT group and 69.2% in the SMT plus GWPT group. The difference was statistically insignificant (p = 1.0), indicating that while both regimens were moderately effective in eliminating *H. pylori*, the addition of GWPT did not produce a significant advantage in microbiological eradication within the two-week follow-up period (p = 1.00) (Table 3).

**Table 3:** Comparison between baseline and two weeks after treatment, endoscopy findings based on rapid urea test in the SMT and SMT plus GWPT groups. Two sample t-test, chi-square test, and Fisher’s exact tests are used to test the independence of attributes. P-value was considered significant at <0.05. Overall eradication rate = 68% across both groups. SMT: standard medical therapy, GWPT: Ganga water-phage therapy

		Group						p-value
		Total		SMT group		SMT plus GWPT group		
		N	%	N	%	N	%	
Rapid urease test								
Baseline visit 2 (n = 50)	Positive	50	100.0%	24	100.0%	26	100.0%	N/A
After two weeks (n = 41)	Negative	34	68.0%	16	66.7%	18	69.2%	1.000

This suggests that GWPT achieved comparable eradication to SMT alone while maintaining excellent safety and tolerability.

Follow-up RUT was available in 41 patients. Eradication was achieved in 68% overall - 16 of 24 (66.7%) in the SMT arm and 18 of 26 (69.2%) in the SMT + GWPT arm (Table 3). The difference was not statistically significant (p = 1.00), indicating comparable microbiological response between regimens

Symptom improvement

This section describes the baseline Global Overall Symptom (GOS) severity distribution among participants in the SMT and SMT plus GWPT groups across multiple dyspeptic domains. For burping, symptoms ranged from mild (36.0% overall) to severe (32.0%), with no significant group differences (p = 0.936). In early satiety, about one-third of participants in each group reported mild problems (33.3% vs. 34.6%), while moderate severity was more frequent in the SMT plus GWPT group (42.3% vs. 29.2%), although not statistically significant (p = 0.540). Regarding the feeling of fullness, most patients experienced mild to moderate problems (68% overall), evenly distributed between the two arms (p = 0.882). Heartburn was common, with severe or very severe problems affecting 64% of participants overall, again without a significant difference (p = 0.571). Sensation of reflux of gastric acid showed a trend toward higher severity in the SMT plus GWPT group (53.8% severe vs. 29.2% in SMT), although the difference approached but did not reach significance (p = 0.064). For gastric pain, over 60% reported severe or very severe pain (25.0% vs. 46.2% and 25.0% vs. 26.9% in SMT and SMT plus GWPT, respectively), with no statistical difference (p = 0.458). Stomach feeling heavy was frequent, with nearly half reporting severe problems (53.8% in SMT plus GWPT vs. 20.8% in SMT), although not significant (p = 0.193). Finally, in feeling queasy, the majority reported moderate severity (56.0% overall), particularly in the SMT plus GWPT group (69.2% vs. 41.7%), with a trend toward significance (p = 0.065). Overall, baseline symptom burdens were high and broadly comparable between groups, with some domains showing non-significant trends toward greater severity in the SMT plus GWPT arm (Table 4).

**Table 4:** GOS severity in the SMT and SMT plus GWPT groups. Two-sample t-test, chi-square test, and Fisher’s exact tests are used to test the independence of attributes. P-value was considered significant at <0.05. SMT: standard medical therapy, GWPT: Ganga water-phage therapy

		Group						p-value
		Total		SMT group		SMT plus GWPT group		
		N	%	N	%	N	%	
Baseline_GOS_Burping	Mild problem	18	36.0%	9	37.5%	9	34.6%	0.936
	Moderate problem	8	16.0%	3	12.5%	5	19.2%	
	No problem	8	16.0%	4	16.7%	4	15.4%	
	Severe problem	16	32.0%	8	33.3%	8	30.8%	
Baseline_GOS_Early satiety	Mild problem	17	34.0%	8	33.3%	9	34.6%	0.540
	Moderate problem	18	36.0%	7	29.2%	11	42.3%	
	No problem	8	16.0%	4	16.7%	4	15.4%	
	Severe problem	7	14.0%	5	20.8%	2	7.7%	
Baseline_GOS_Feeling of fullness	Mild problem	21	42.0%	11	45.8%	10	38.5%	0.882
	Moderate problem	13	26.0%	5	20.8%	8	30.8%	
	No problem	6	12.0%	3	12.5%	3	11.5%	
	Severe problem	10	20.0%	5	20.8%	5	19.2%	
Baseline_GOS_Heartburn	Mild problem	8	16.0%	6	25.0%	2	7.7%	0.571
	Moderate problem	5	10.0%	2	8.3%	3	11.5%	
	No problem	5	10.0%	2	8.3%	3	11.5%	
	Severe problem	17	34.0%	7	29.2%	10	38.5%	
	Very severe problem	15	30.0%	7	29.2%	8	30.8%	
Baseline_GOS_Sensation of reflux of gastric acid	Mild problem	6	12.0%	5	20.8%	1	3.8%	0.064
	Moderate problem	8	16.0%	3	12.5%	5	19.2%	
	No problem	6	12.0%	2	8.3%	4	15.4%	
	Severe problem	21	42.0%	7	29.2%	14	53.8%	
	Very severe problem	9	18.0%	7	29.2%	2	7.7%	
Baseline_GOS_Gastric pain	Mild problem	7	14.0%	4	16.7%	3	11.5%	0.458
	Moderate problem	7	14.0%	5	20.8%	2	7.7%	
	No problem	5	10.0%	3	12.5%	2	7.7%	
	Severe problem	18	36.0%	6	25.0%	12	46.2%	
	Very severe problem	13	26.0%	6	25.0%	7	26.9%	
Baseline_GOS_Stomach feeling heavy	Mild problem	9	18.0%	6	25.0%	3	11.5%	0.193
	Moderate problem	13	26.0%	8	33.3%	5	19.2%	
	No problem	4	8.0%	2	8.3%	2	7.7%	
	Severe problem	19	38.0%	5	20.8%	14	53.8%	
	Very severe problem	5	10.0%	3	12.5%	2	7.7%	
Baseline_GOS_Feeling queasy	Mild problem	4	8.0%	2	8.3%	2	7.7%	0.065
	Moderate problem	28	56.0%	10	41.7%	18	69.2%	
	No problem	5	10.0%	3	12.5%	2	7.7%	
	Severe problem	4	8.0%	1	4.2%	3	11.5%	
	Very severe problem	9	18.0%	8	33.3%	1	3.8%	

At baseline, most participants reported moderate-to-severe dyspeptic symptoms across multiple domains (Table 4). After two weeks of therapy, both groups showed marked improvement, with higher proportions of symptom-free (“no problem”) patients in the SMT + GWPT group across all domains.

For example, symptom-free status for burping improved from 16% → 61.5% in the SMT + GWPT group versus 16.7% → 41.7% in SMT alone; for early satiety, 15.4% → 65.4% in the GWPT arm compared to 16.7% → 45.8%.

Although inter-group differences were not statistically significant (all p > 0.05), the adjunct group consistently demonstrated greater symptomatic relief in nearly every domain, suggesting a favorable clinical trend.

Table 5 illustrates the GOS severity distribution two weeks after treatment in the SMT and SMT plus GWPT groups. Across all domains, both groups showed symptomatic improvement, with a higher proportion of “no problem” (symptom-free) patients in the SMT plus GWPT arm, although differences were not statistically significant. For burping, 61.5% of patients in the SMT plus GWPT group reported complete resolution compared to 41.7% in the SMT group (p = 0.537). In early satiety, 65.4% vs. 45.8% achieved symptom-free status (p = 0.332), while for the feeling of fullness, 61.5% vs. 45.8% reported resolution (p = 0.204). Similarly, heartburn improved in 65.4% of SMT plus GWPT patients compared to 50.0% in SMT (p = 0.277). For the sensation of reflux of gastric acid, symptom-free rates were 53.8% vs. 37.5% (p = 0.207). In gastric pain, 53.8% of the SMT plus GWPT group reported no problem compared with 37.5% of the SMT group (p = 0.336). Stomach feeling heavy improved in 53.8% of SMT plus GWPT participants versus 37.5% in SMT (p = 0.113), and for feeling queasy, 53.8% vs. 29.2% were symptom-free (p = 0.082). Across all symptom domains, the adjunct GWPT group consistently demonstrated higher frequencies of resolution and lower frequencies of very severe symptoms, though none of the differences reached statistical significance. This suggests a trend toward better symptomatic relief with GWPT as an adjunct to standard therapy (Table 5).

**Table 5:** GOS severity at two-week post-treatment in the SMT and SMT plus GWPT groups. Two-sample t-test, chi-square test, and Fisher’s exact tests are used to test the independence of attributes. P-value was considered significant at <0.05. SMT: standard medical therapy, GWPT: Ganga water-phage therapy

After 2 Weeks_GOS_Burping	Mild problem	9	18.0%	6	25.0%	3	11.5%	0.537
	Moderate problem	4	8.0%	2	8.3%	2	7.7%	
	No problem	26	52.0%	10	41.7%	16	61.5%	
	Very severe problem	2	4.0%	1	4.2%	1	3.8%	
After 2 Weeks_GOS_Early satiety	Mild problem	3	6.0%	2	8.3%	1	3.8%	0.332
	Moderate problem	5	10.0%	2	8.3%	3	11.5%	
	No problem	28	56.0%	11	45.8%	17	65.4%	
	Very severe problem	5	10.0%	4	16.7%	1	3.8%	
After 2 Weeks_GOS_Feeling of fullness	Mild problem	6	12.0%	2	8.3%	4	15.4%	0.204
	Moderate problem	5	10.0%	3	12.5%	2	7.7%	
	No problem	27	54.0%	11	45.8%	16	61.5%	
	Very severe problem	3	6.0%	3	12.5%	0	0.0%	
After 2 Weeks_GOS_Heartburn	Mild problem	5	10.0%	2	8.3%	3	11.5%	0.277
	Moderate problem	4	8.0%	2	8.3%	2	7.7%	
	No problem	29	58.0%	12	50.0%	17	65.4%	
	Very severe problem	3	6.0%	3	12.5%	0	0.0%	
After 2 Weeks_GOS_Sensation of reflux of gastric acid	Mild problem	12	24.0%	5	20.8%	7	26.9%	0.207
	Moderate problem	3	6.0%	2	8.3%	1	3.8%	
	No problem	23	46.0%	9	37.5%	14	53.8%	
	Very severe problem	3	6.0%	3	12.5%	0	0.0%	
After 2 Weeks_GOS_Gastric pain	Mild problem	8	16.0%	3	12.5%	5	19.2%	0.336
	Moderate problem	2	4.0%	1	4.2%	1	3.8%	
	No problem	23	46.0%	9	37.5%	14	53.8%	
	Very severe problem	8	16.0%	6	25.0%	2	7.7%	
After 2 Weeks_GOS_Stomach feeling heavy	Mild problem	13	26.0%	7	29.2%	6	23.1%	0.113
	Moderate problem	3	6.0%	3	12.5%	0	0.0%	
	No problem	23	46.0%	9	37.5%	14	53.8%	
	Very severe problem	2	4.0%	0	.0%	2	7.7%	
After 2 Weeks_GOS_Feeling queasy	Mild problem	11	22.0%	5	20.8%	6	23.1%	0.082
	No problem	21	42.0%	7	29.2%	14	53.8%	
	Very severe problem	9	18.0%	7	29.2%	2	7.7%	
After 2 Weeks_GOS_Burping	Mild problem	9	18.0%	6	25.0%	3	11.5%	0.537
	Moderate problem	4	8.0%	2	8.3%	2	7.7%	
	No problem	26	52.0%	10	41.7%	16	61.5%	
	Very severe problem	2	4.0%	1	4.2%	1	3.8%	
After 2 Weeks_GOS_Early satiety	Mild problem	3	6.0%	2	8.3%	1	3.8%	0.332
	Moderate problem	5	10.0%	2	8.3%	3	11.5%	
	No problem	28	56.0%	11	45.8%	17	65.4%	
	Very severe problem	5	10.0%	4	16.7%	1	3.8%	
After 2 Weeks_GOS_Feeling of fullness	Mild problem	6	12.0%	2	8.3%	4	15.4%	0.204
	Moderate problem	5	10.0%	3	12.5%	2	7.7%	
	No problem	27	54.0%	11	45.8%	16	61.5%	
	Very severe problem	3	6.0%	3	12.5%	0	0.0%	
After 2 Weeks_GOS_Heartburn	Mild problem	5	10.0%	2	8.3%	3	11.5%	0.277
	Moderate problem	4	8.0%	2	8.3%	2	7.7%	
	No problem	29	58.0%	12	50.0%	17	65.4%	
	Very severe problem	3	6.0%	3	12.5%	0	0.0%	
After 2 Weeks_GOS_Sensation of reflux of gastric acid	Mild problem	12	24.0%	5	20.8%	7	26.9%	0.207
	Moderate problem	3	6.0%	2	8.3%	1	3.8%	
	No problem	23	46.0%	9	37.5%	14	53.8%	
	Very severe problem	3	6.0%	3	12.5%	0	0.0%	
After 2 Weeks_GOS_Gastric pain	Mild problem	8	16.0%	3	12.5%	5	19.2%	0.336
	Moderate problem	2	4.0%	1	4.2%	1	3.8%	
	No problem	23	46.0%	9	37.5%	14	53.8%	
	Very severe problem	8	16.0%	6	25.0%	2	7.7%	
After 2 Weeks_GOS_Stomach feeling heavy	Mild problem	13	26.0%	7	29.2%	6	23.1%	0.113
	Moderate problem	3	6.0%	3	12.5%	0	0.0%	
	No problem	23	46.0%	9	37.5%	14	53.8%	
	Very severe problem	2	4.0%	0	.0%	2	7.7%	
After 2 Weeks_GOS_Feeling queasy	Mild problem	11	22.0%	5	20.8%	6	23.1%	0.082
	No problem	21	42.0%	7	29.2%	14	53.8%	
	Very severe problem	9	18.0%	7	29.2%	2	7.7%	

Quality of life

This section presents the comparison of mean SF-36 quality-of-life domain scores between the SMT and SMT plus GWPT groups at two weeks post-treatment. Overall, both groups demonstrated improvements, with the SMT plus GWPT arm showing numerically higher gains in several domains, although differences were not statistically significant. Physical function scores were similar (19.08 ± 2.39 vs. 18.54 ± 3.94, p = 0.557), as were social function (6.21 ± 1.77 vs. 6.23 ± 1.56, p = 0.658) and physical health problems (5.00 ± 1.53 vs. 5.15 ± 1.01, p = 0.182). Emotional problems showed a trend toward better outcomes in the SMT plus GWPT group (3.69 ± 0.84 vs. 4.25 ± 1.15, p = 0.078), although not statistically significant. Similarly, mental health (20.29 ± 5.68 vs. 17.62 ± 2.25, p = 0.191) and vitality (14.75 ± 5.67 vs. 12.77 ± 2.70, p = 0.446) indicated modest improvements in both arms. The pain domain reflected slightly lower scores in SMT plus GWPT (6.96 ± 1.66 vs. 6.13 ± 2.31, p = 0.440), suggesting better pain relief, while overall evaluation of health was also slightly higher in the GWPT group (20.27 ± 2.20 vs. 19.62 ± 2.30, p = 0.313). Taken together, these results indicate that while inter-group differences did not reach statistical significance, the SMT plus GWPT group demonstrated a consistent trend toward better quality-of-life outcomes across physical, emotional, and symptomatic domains, complementing the symptomatic relief observed in GOS assessments (Table 6).

**Table 6:** Comparison between the SMT and SMT plus GWPT groups for mean SF-36 domain scores. Applied Mann-Whitney U test

	Group						Z value	p-value
	SMT group		SMT plus GWPT group		Total			
	Mean	SD	Mean	SD	Mean	SD		
Physical function	19.08	2.39	18.54	3.94	18.80	3.27	-0.587	0.557
Social function	6.21	1.77	6.23	1.56	6.22	1.64	-0.443	0.658
Physical health problems	5.00	1.53	5.15	1.01	5.08	1.28	-1.335	0.182
Emotional problems	4.25	1.15	3.69	.84	3.96	1.03	-1.763	0.078
Mental health	20.29	5.68	17.62	2.25	18.90	4.42	-1.308	0.191
Vitality	14.75	5.67	12.77	2.70	13.72	4.45	-0.762	0.446
Pain	6.13	2.31	6.96	1.66	6.56	2.02	-0.773	0.440
Overall evaluation of health	19.62	2.30	20.27	2.20	19.96	2.25	-1.01	0.313

Quality-of-life assessment (SF-36) showed improvement in most domains for both groups (Table 6). Between-group differences were not statistically significant; however, within-group analysis revealed significant gains in the SMT + GWPT arm in physical function, emotional well-being, vitality, pain, and overall health perception (all p < 0.05).

The SMT arm showed modest improvement, limited to physical and emotional domains. The greater magnitude of change in the GWPT arm supports enhanced subjective recovery and overall well-being.

**Figure 2:**
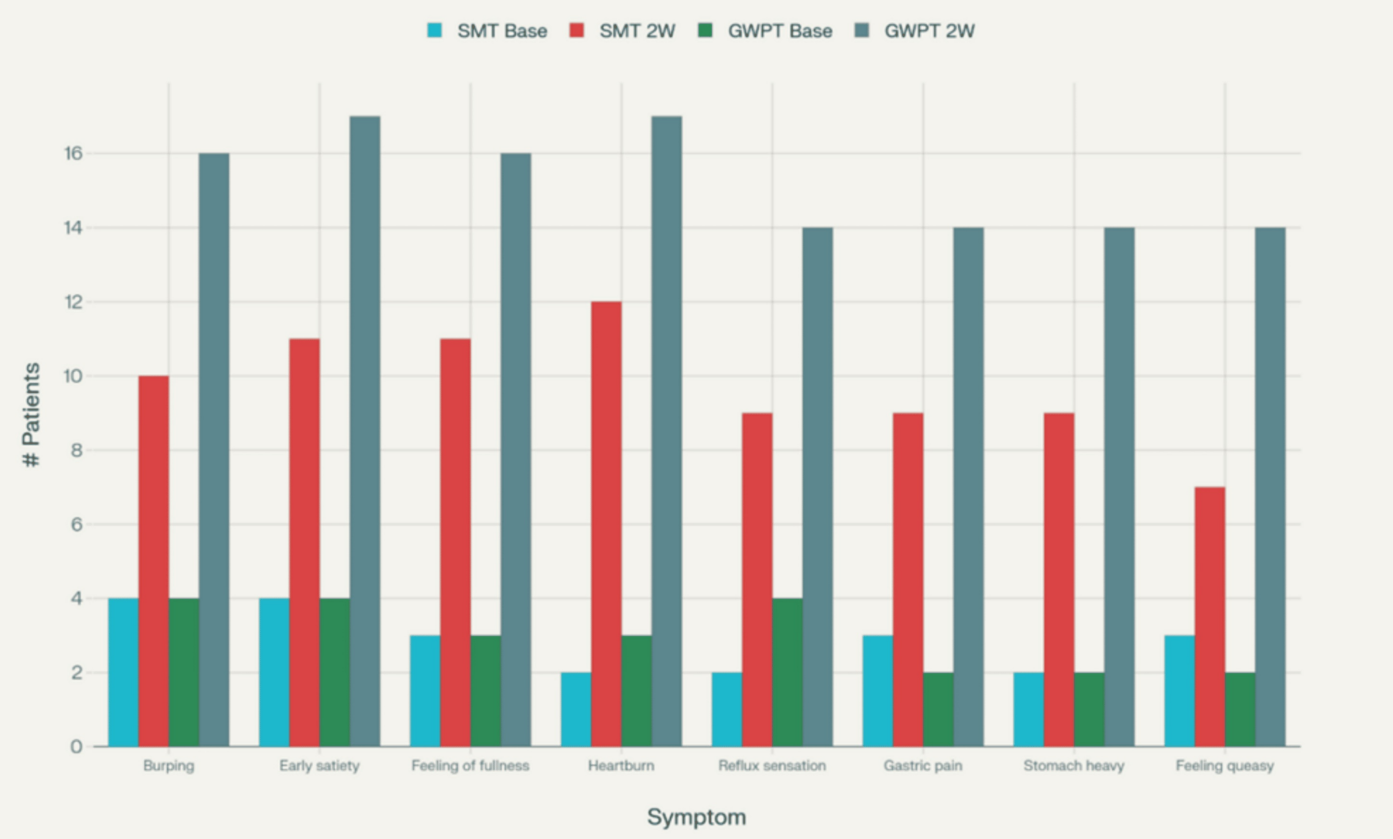
Graphical representation: no problem (symptom-free) patients Comparison of "no problem" (symptom-free) counts by group and timepoint.

At two weeks post-treatment, both groups demonstrated improvements in SF-36 quality-of-life scores, with the SMT plus GWPT group showing more robust and consistent gains. In the SMT group, significant improvements were observed in physical function, physical health problems, and emotional problems, while changes in mental health, vitality, pain, and overall health perception were modest and statistically nonsignificant. By contrast, the SMT plus GWPT group showed significant improvements across multiple domains, including physical function, physical health problems, emotional problems, mental health, vitality, pain relief, and overall evaluation of health, with only social function remaining unchanged. Between-group comparisons revealed no statistically significant differences; however, the SMT plus GWPT group consistently demonstrated greater mean improvements across most domains, particularly in physical function, emotional well-being, vitality, and pain reduction, suggesting an added quality-of-life benefit from the adjunct therapy (Table 7). The comparative changes in the count of completely symptom-free (‘no problem’) patients by group and time point are graphically represented in Figure 2.

**Table 7:** Comparison between changes in SF-36 domain scores from baseline to two weeks after treatment in the SMT and SMT plus GWPT groups Applied the Wilcoxon signed-rank test for significance

			Paired samples' statistics						
Group			Mean	N	SD	Mean diff.	%Mean change	Z value	p-value
SMT group	Physical function	Visit 2	19.74	19	2.08	-3.32	-16.80	-2.667	0.008
		After two weeks	23.05	19	3.85				
	Social function	Visit 2	6.37	19	1.83	0.95	14.88	-1.259	0.208
		After two weeks	5.42	19	1.57				
	Physical health problems	Visit 2	5.05	19	1.51	-1.89	-37.50	-3.232	0.001
		After two weeks	6.95	19	0.85				
	Emotional problems	Visit 2	4.42	19	1.22	-0.79	-17.86	-2.276	0.023
		After two weeks	5.21	19	0.54				
	Mental health	Visit 2	20.79	19	6.28	1.58	7.60	-.916	0.360
		After two weeks	19.21	19	4.39				
	Vitality	Visit 2	15.63	19	6.08	-1.32	-8.42	-.786	0.432
		After two weeks	16.95	19	2.95				
	Pain	Visit 2	5.79	19	2.42	-0.16	-2.73	-.378	0.706
		After two weeks	5.95	19	2.57				
	Overall evaluation of health	Visit 2	19.42	19	2.24	0.63	3.25	-1.066	0.286
		After two weeks	18.79	19	2.18				
SMT plus GWPT group	Physical function	Visit 2	18.23	22	3.42	-8.86	-48.63	-4.030	<0.001
		After two weeks	27.09	22	2.67				
	Social function	Visit 2	6.14	22	1.64	-0.14	-2.22	-.123	0.902
		After two weeks	6.27	22	0.94				
	Physical health problems	Visit 2	5.05	22	0.84	-2.50	-49.55	-4.169	<0.001
		After two weeks	7.55	22	0.51				
	Emotional problems	Visit 2	3.55	22	0.74	-2.18	-61.54	-4.091	<0.001
		After two weeks	5.73	22	0.55				
	Mental health	Visit 2	17.23	22	2.16	-2.36	-13.72	-2.304	0.021
		After two weeks	19.59	22	3.39				
	Vitality	Visit 2	12.82	22	2.70	-3.36	-26.24	-3.163	0.002
		After two weeks	16.18	22	3.23				
	Pain	Visit 2	7.18	22	1.68	3.00	41.77	-3.613	<0.001
		After two weeks	4.18	22	1.94				
	Overall evaluation of health	Visit 2	20.23	22	2.31	4.05	20.00	-3.630	<0.001
		After two weeks	16.18	22	2.87				

Safety

**Figure 3:**
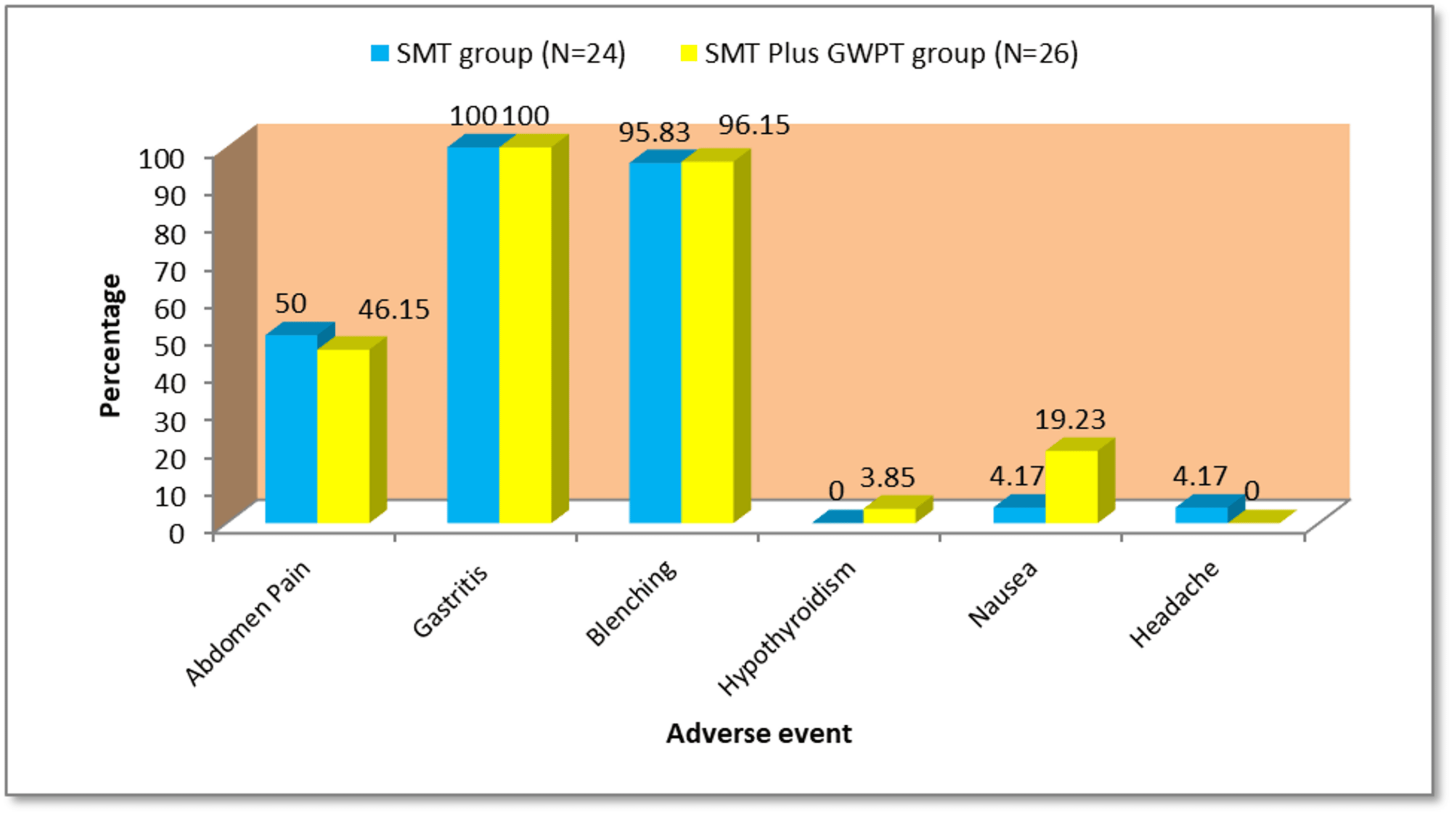
Frequency of adverse events in the SMT and SMT plus GWPT groups SMT: standard medical therapy, GWPT: Ganga water-phage therapy

Adverse events were frequent but mild, including abdominal pain, gastritis, and belching in both groups. Nausea was slightly higher in SMT+GWPT. One case of hypothyroidism occurred (Figure 3).

Mild adverse events such as abdominal discomfort, nausea, belching, and transient gastritis occurred in both groups, mostly CTCAE grade 1. No serious or grade ≥3 events were reported. Event frequencies were similar except for slightly higher nausea in the GWPT arm. One isolated case of hypothyroidism was judged unrelated to study therapy (Figure 3). Overall tolerability was good.

## Discussion

In this randomized comparative study, both treatment arms (SMT and SMT plus GWPT) were well matched at baseline for demographic, lifestyle, and laboratory characteristics, reducing confounding and supporting the validity of outcome comparisons. At baseline, all participants were positive for RUT, confirming *H. pylori* infection. After two weeks, overall, RUT negativity was 68%, with 66.7% in SMT and 69.2% in SMT plus GWPT. GWPT plus SMT did not significantly enhance eradication (p = 1.000) but was associated with improved symptom relief and quality-of-life scores. These findings are consistent with earlier Indian reports. Ray et al. observed 53% eradication in Puducherry [15]. Agarwal et al. reported up to 85% in northern India [16], and Paul et al. documented 36% in southern Kerala [17]. This variability between regions reflects different patterns of antimicrobial resistance, as highlighted by Datta et al. [18], who reported increased resistance to clarithromycin and metronidazole in Indian centers. Our study adds further evidence from northern India, demonstrating moderate efficacy of standard triple therapy and showing that adjunct GWPT may improve *H. pylori* eradication without statistical significance.

Previous works have linked the therapeutic effects of Ganga water to naturally occurring bacteriophages with antibacterial activity [19-20] and historical observations of antimicrobial properties [14]. As with our findings, Shukla et al. (2021) also reported symptomatic benefits of Ganga water in dyspepsia [11]. These results strengthen the hypothesis that naturally derived phages may offer supplementary benefits to conventional therapy, aligning with the concept of microbiota modulation rather than direct bacterial elimination. Our findings are also in line with the international orientation of the Toronto Consensus [21] and the Maastricht/Florence Consensus [22], which recommend adapting therapy according to regional antimicrobial resistance profiles and exploring adjunctive or alternative strategies in areas of high clarithromycin resistance.

The initial load of symptoms was high, with gastric pain and frequent reflux. After two weeks, both groups showed clinically significant improvement, with more participants reporting symptom resolution in all GOSS domains. Although the differences between the groups were not statistically significant, the SMT plus GWPT arm consistently demonstrated numerically higher proportions of symptom-free patients, suggesting a potential adjuvant effect of GWPT. Similarly, SF-36 scores in follow-up were comparable between the groups, but within-group analysis revealed broader and more significant gains in the SMT plus GWPT arm across physical, emotional, and vitality domains, as well as pain reduction. This indicates that GWPT may have contributed to better patient-reported outcomes and subjective well-being. Such symptomatic benefits without marked eradication differences have also been observed with other non-antibiotic adjuvants, including Lactobacillus reuteri, Saccharomyces boulardii, and herbal agents such as Nigella sativa, which improved tolerability and gastric comfort despite modest effects on bacterial clearance.

Adverse events were frequent but usually mild, without severe or treatment-limiting effects. Dyspeptic complaints such as abdominal pain, gastritis, and belching were common in both groups, while nausea was slightly more frequent in the GWPT arm, and a single case of hypothyroidism occurred. Overall tolerability was good, suggesting that GWPT can be safely administered alongside SMT. Safety monitoring was conducted using the Common Terminology Criteria for Adverse Events (CTCAE v5.0) framework, ensuring standardized risk assessment and consistent documentation. No major deviations or environmental safety issues were reported, confirming compliance with biosafety and environmental ethics standards as per CPCB and ICMR guidelines.

Our rationale was based on the concept of Ganga-based adjuvant therapy (GWPT), paralleling historical observations of the therapeutic potential of Ganga water [23] and modern findings of bacteriophages within these waters [24, 25]. The study of Alaknanda in Rudraprayag [26] reported favorable physicochemical parameters (pH 7.68, turbidity < NTU, TDS 140 mg/L), absence of coliform contamination, and isolation of non-pathogenic bacteria such as Micrococcus. Although trace metals such as aluminum, iron, and lead were present, they remained within permissible limits [26]. These findings confirm that the upper flow of Ganga/Alaknanda water is microbiologically safe, aligning with our biosafety screening that confirmed the absence of pathogenic contamination before participant use.

The effects of GWPT in our study, particularly in symptom relief and quality of life, may partially reflect these physical and microbiological properties. Shukla et al. [11] further demonstrated clinical benefits in dyspepsia, complementing environmental studies and showing that a structured GWPT regimen, when combined with SMT, improves patient-centered outcomes without compromising safety.

Limitations

The strengths of our study include its randomized design, well-matched baseline characteristics, and the holistic evaluation of microbiological, symptomatic, and quality-of-life outcomes. Limitations include a small sample size, a short two-week follow-up, reliance on RUT rather than confirmatory diagnostics such as the ^13^C-urea breath test or biopsy [27], and the absence of antimicrobial resistance profiling. The open-label design, though pragmatic for a preliminary human trial, introduces potential bias in subjective outcomes. Furthermore, as GWPT was delivered as an adjunct, its independent effect cannot be fully separated. Nonetheless, our safety results demonstrate ethical and biosafety compliance, and no participant incentives or conflicts of interest were involved.

These findings are consistent with Indian eradication trials reporting moderate efficacy [2,3,6,16] and reinforce international guidance advocating for alternative or adjunct regimens in high-resistance settings [17, 18]. Together with environmental studies confirming the microbiological safety of Himalayan Ganga water [22], our results suggest that adjunctive therapies such as GWPT may improve patient-centered outcomes in dyspepsia and *H. pylori* infection.

Clinical implications

The findings of this study have several implications for clinical practice. First, GWPT did not significantly enhance *H. pylori *eradication but showed potential to modestly improve outcomes when combined with standard triple therapy. Second, it consistently demonstrated symptomatic and quality-of-life benefits, suggesting value as an adjuvant approach in dyspepsia management, particularly in contexts where bacterial eradication alone does not ensure patient satisfaction. Third, the good tolerability and absence of serious adverse events further support the safety of integrating GWPT with standard regimens. Given the increasing burden of antimicrobial resistance and the variable success of eradication across regions, such adjunctive options that enhance patient-centered outcomes without adding risk are clinically relevant. Finally, this study underscores the importance of integrating therapies that address both microbiological and symptomatic aspects of dyspepsia, aligning with global recommendations for holistic care. GWPT thus represents a safe, culturally acceptable, and potentially effective support therapy. However, larger multicentric studies with longer follow-up and standardized molecular characterization of phage activity are warranted before its widespread clinical application.

## Conclusions

In summary, GWPT combined with SMT did not significantly enhance *H. pylori *eradication but was associated with improved symptom relief and quality of life. It needs further study as to whether (1) GWPT alone may be effective and (2) variation in doses of GWPT may beget better results. These results suggest that the combination of adjuvant therapies, such as GWPT with optimized eradication regimes, can improve patient-centered results in dyspepsia and H. pylori infection. Larger and longer studies with robust diagnoses and resistance mapping are required.
